# Risk Assessment of Alien Woody Plants in China’s National Nature Reserves Under Climate Change

**DOI:** 10.3390/plants14193006

**Published:** 2025-09-28

**Authors:** Da-Zhi Wang, Chun-Jing Wang, Fei-Xue Zhang, Hong-Li Li

**Affiliations:** 1 School of Ecology and Nature Conservation, Beijing Forestry University, Beijing 100083, China; wangdazhi1212@163.com (D.-Z.W.); zfx660109@163.com (F.-X.Z.); 2 The Key Laboratory of Ecological Protection in the Yellow River Basin of National Forestry and Grassland Administration, School of Ecology and Nature Conservation, Beijing Forestry University, Beijing 100083, China; 3 Sichuan Academy of Forestry, Chengdu 610081, China; wangchunjing00@163.com

**Keywords:** alien woody plants, climate change, national nature reserves, MaxEnt modeling, species distribution modeling

## Abstract

Alien woody plants (AWPs) increasingly threaten biodiversity in China’s national nature reserves, with climate change expected to intensify these risks. We used species distribution modeling (MaxEnt) and spatial prioritization (Zonation) to assess invasion risk for 251 AWP species across 479 national nature reserves under current and future climate scenarios (SSP245 and SSP585). Spatial prioritization revealed current hotspots in southern tropical–subtropical national nature reserves (e.g., Hainan, Fujian, Yunnan provinces), with significant northward and westward expansion projected under warming. A total of 71 species—such as *Quercus robur*, *Salix alba*, and *Robinia pseudoacacia*—pose consistently high risks, while some others (e.g., *Ficus benghalensis*) may become emerging threats under future conditions. These range shifts are driven by thermal constraint relaxation and longer growing seasons. To mitigate future impacts, we recommend region-specific strategies: containment and seed-source control in southern national nature reserves, and early detection and monitoring in northern and western regions. Our findings provide a spatially explicit framework for climate-informed invasive species management in protected areas.

## 1. Introduction

Plant invasion is the process by which alien plant species spread rapidly and establish stable populations in their non-native habitats, negatively affecting local biodiversity and ecosystem function [1]. Alien woody plants (AWP) pose a profound and persistent threat to terrestrial ecosystems, and their longevity, structural dominance and ability to alter ecosystem functioning make them distinct from other invasive species forms [2]. Unlike herbaceous invaders, invasive trees often trigger cascading effects from canopy dominance to soil legacy effects [3] and its deep root system may disrupt the hydrologic cycle [4]. These invasions often lead to biodiversity loss in protected areas, as AWPs outcompete native species for resources such as light, water, and nutrients [5]. Additionally, they degrade ecosystem services critical for human well-being, including water regulation, carbon sequestration, and soil stabilization [6].

Climate change is reshaping global biodiversity patterns and invasive species are both drivers and beneficiaries of ecosystem change. Warmer temperatures and changes in precipitation are expanding the potential ecological niche for non-native plants, especially woody invasive species, which threaten native forest ecosystems through competitive exclusion, disruption of the carbon cycle, and degradation of ecosystem services (e.g., water regulation and soil conservation) [7]. Climate change can intensify woody plant invasions worldwide by reshaping ecosystem conditions, including temperature, precipitation regimes, and seasonality [8,9]. Such shifts create more favorable environments for AWPs, influencing their growth and distribution [10]. For instance, warmer climates enhance survival and reproduction, enabling species previously constrained by low temperatures to expand into higher latitudes or colder regions [11]. Warming will result in a longer growing season, allowing AWP to grow and reproduce further [12]. These changes have not only altered the distribution of species, but also exacerbated pressures on protected areas, traditionally seen as strongholds of biodiversity, which now face the dual challenge of mitigating climate impacts while resisting homogenization [13]. However, the synergistic effects of climate change and biological invasions on these critical habitats remain under-quantified, especially in biodiversity-rich regions such as China, where complex topography and diverse climates create unique opportunities for invasive species to take advantage of changing environmental conditions.

China is one of the richest countries in the world in terms of biodiversity and also a hotspot for biological invasions [14]. China’s unique geographic and climatic features—such as its broad latitudinal span ranging from tropical to cold-temperate zones—contribute to its exceptionally high biodiversity [15]. However, this biogeographic advantage also makes China highly vulnerable to the twin threats of climate change and biological invasions [16]. For example, the rate of invasion of AWPs in China has accelerated significantly in recent decades with the intensification of international trade and climate warming. Some of these species pose serious threats to the stability of native ecosystems [16]. As a cornerstone of biodiversity conservation, a nature reserve is legally defined as an area designated for the special protection and management of representative natural ecosystems; natural concentrated distribution areas of rare and endangered wildlife and plant species; and land, water, or sea areas housing natural relics of special significance [17]. These natural reserves provide essential habitat for endangered species, such as giant pandas and northeastern tigers [18,19]. Additionally, they play a crucial role in sustaining ecosystem services [17,20].

While earlier research has identified region-specific challenges of AWP invasions—such as elevated invasion risks in northern China and stronger competition in the south—systematic and comprehensive evaluations of these risks across national nature reserves under current and future climate scenarios remain scarce [21,22]. This study addresses this critical knowledge gap by providing the first large-scale, spatially explicit evaluation of invasion dynamics for 251 alien woody species across all national nature reserves in China under multiple climate change scenarios. By integrating species distribution models with conservation prioritization tools, our research offers novel insights into how climate change differentially affects woody plant invasions across biogeographical regions. These findings are essential for informing adaptive management strategies and serve as a scientific reference for biodiversity conservation and invasive species control in global biodiversity hotspots.

This study aims to answer the two questions:(1) Which regions in China’s national nature reserves are at the highest risk of AWP invasion under current and future climates? and (2) Which AWP species pose the greatest threats to these protected areas? This study can reveal the combined effects of climate change and invasion of alien woody plants, fill the gap of multi-species and multi-scenario invasion risk assessment in China, and provide quantitative basis for the development of climate.

## 2. Results

### 2.1. Species Distribution Patterns and Trends in the Context of Climate Change

**Figure 1:**
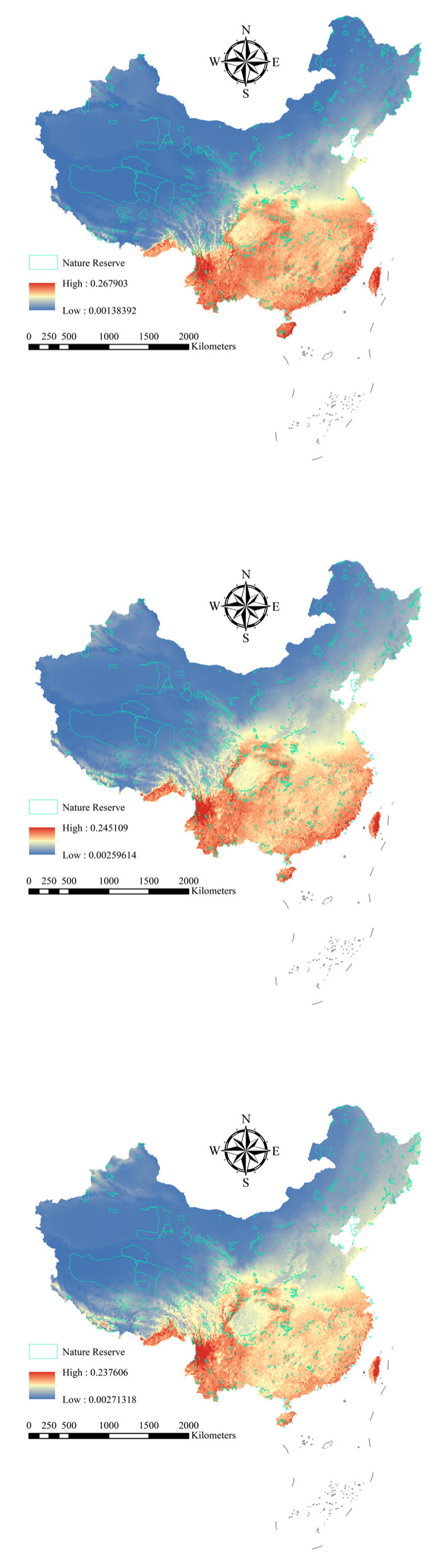
Average distribution probability of alien woody plants under different climate scenarios (current, SSP245, and SSP585). Red, yellow, and blue represent high, medium, and low probabilities of plant species distributions, respectively.

**Figure 2:**
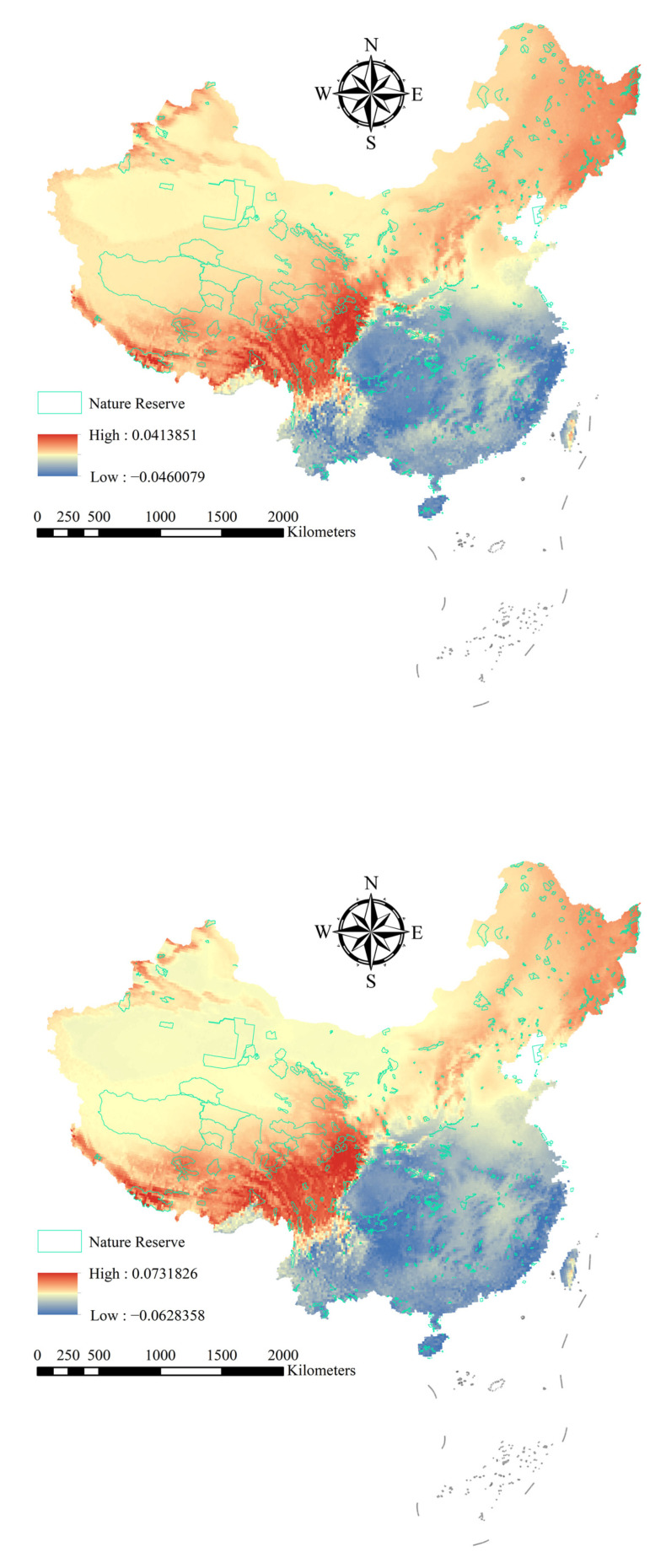
Change in the potential distribution of alien woody plants between current and future distributions. Red, yellow, and blue represent high, medium, and low probabilities of plant species distributions, respectively.

For the 251 Chinese alien woody plants, the average AUC was 0.909 (±0.071 standard deviation [SD]) for training data and 0.907 (±0.070 SD) for test data (Table S1, Figure S1). The results showed that the maxent model performed well, and all the results are applicable to further experiments involving Chinese alien woody plants. Under current and future climatic conditions, the high distribution areas of the 251 Chinese alien woody plants are concentrated in Hainan, Fujian, Zhejiang and Yunnan provinces (Figure 1). However, with increasing climate change, the alien woody plants will expand to the north (Figure 2).

Under the current climatic conditions, the average distribution probability alien woody plants of in national nature reserves shows obvious regional differences. The average distribution probability of alien woody plants in nature reserves in southern provinces such as Hainan is generally high (Figure 1), especially in nature reserves such as Diaoluoshan National Nature Reserve (Average distribution probability = 0.227), Zhangjiangkou Mangrove Forest National Nature Reserve (average distribution probability = 0.214), Wuzhishan National Nature Reserve (average distribution probability = 0.213), Dongzhai Harbor National Nature Reserve (average distribution probability = 0.201) and Cangshan Erhai National Nature Reserve (average distribution probability = 0.201). Some nature reserves in the central and eastern regions have intermediate distribution probabilities. The distribution probability of nature reserves in the northern and western regions is low.

However, under the future climate scenarios (SSP245 and SSP585), the distribution probability of alien woody plants increases significantly in national nature reserves in the northern and western regions (Figure 2). For example, the distribution probability of invasive woody plant species in national nature reserves in the northern region, such as Heilongjiang Langxiang National Nature Reserve and Heilongjiang Qixing Lazi Northeast Tiger National Nature Reserve, showed an increasing trend. Meanwhile, the distribution probability of invasive woody plants in nature reserves in western regions, such as Gansu Annanba Wild Camel National Nature Reserve and Ningxia Habahu National Nature Reserve, has also increased (Figure 2). In contrast, the distribution probability of invasive woody plants in national nature reserves in the southern and central regions generally decreased.

To address potential concerns regarding the interpretation of relatively low mean probability values (e.g., ~0.2), we conducted a correlation analysis between our continuous mean probability metric and an alternative risk indicator: the number of species predicted to occur using a binary threshold of 0.5. A strong and statistically significant positive correlation (*r* = 0.90, *p* < 0.001) was found between these two metrics. This high correlation demonstrates that areas identified as high-risk by our continuous probability approach are the same areas identified as high-risk by the binary threshold method. Therefore, our use of mean probability as a risk indicator is statistically robust and effectively captures the spatial pattern of multi-species invasion pressure, validating its application in our study.

### 2.2. Highest Risk Areas and Protected Areas

**Figure 3:**
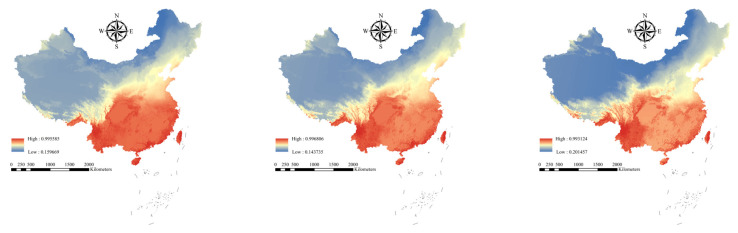
Spatial prioritization of AWP invasion risk in China under current and future climate scenarios (SSP245 and SSP585). Red, yellow, and blue represent high, medium, and low probabilities of plant species distributions, respectively.

Zonation analysis showed that the invasion risk of alien woody plants in China’s national nature reserves exhibited significant spatial differentiation (Figure 3). Under current climate conditions, southern reserves showed the highest invasion risk, concentrated in tropical–subtropical areas such as Hainan and Fujian. In contrast, northern reserves (e.g., Huzhong National Nature Reserve (Heilongjiang)) and western arid zones (e.g., Altun Shan National Nature Reserve (Xinjiang)) exhibited lower risks.

**Figure 4:**
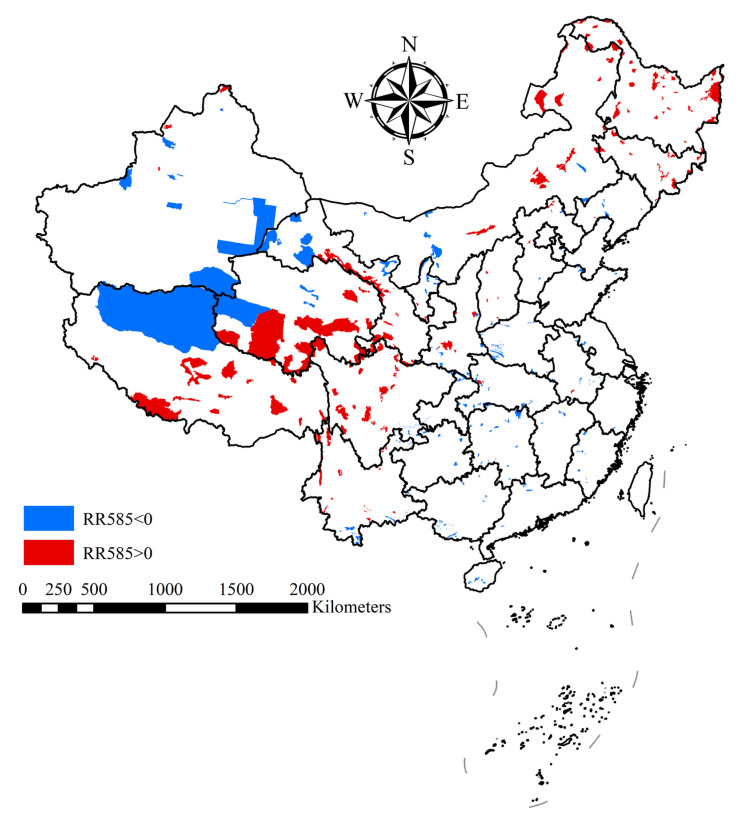
Logarithmic response ratios of alien woody plants in China’s national nature reserves under the SSP585 climate scenario (RR > 0 represents an elevated risk of future intrusion, RR < 0 represents a reduced risk of future intrusion).

Under the SSP245 scenario, the invasion risk increases in northern and western national nature reserves, mainly concentrated in Heilongjiang, Inner Mongolia, and other provinces (Table S2). Examples include Chonar River National Nature Reserve (Heilongjiang; RR245 = 0.315) and Bila River National Nature Reserve (Inner Mongolia; RR245 = 0.294). The SSP585 scenario exacerbates the expansion trend, with heightened risks primarily in Heilongjiang, Inner Mongolia, Qinghai, and Tibet (Figure 4). For instance, the invasion risk in the Chonar River (Heilongjiang; RR585 = 0.436) is further increasing., Selin Co National Nature Reserve (Tibet; RR585 = 0.377) emerges as a new hotspot. While the southern coastal regions maintain high threats, some inland areas exhibit slight risk reduction, such as Honghu National Nature Reserve (Hubei; RR585 = −0.037).

Despite the numerically low average distribution probabilities (e.g., <0.25), these values should not be interpreted as an indicator of low overall risk. The MaxEnt model outputs represent a relative index of environmental suitability rather than an absolute probability of occurrence. In large-scale ecological niche models encompassing vast and environmentally heterogeneous regions like China, absolute suitability values are naturally attenuated. The critical insight lies not in the absolute value, but in the relative spatial patterns and the temporal shifts they reveal. Our identification of high-risk areas and species is based on this relative framework. The designated hotspots (e.g., Diaoluoshan) exhibit suitability scores that are markedly higher than the vast majority of the landscape. Furthermore, the significant northward and westward expansion of suitability, quantified by the positive logarithmic response ratios (RR) in many nature reserves (e.g., RR_585_ > 0.4 for Chonar River), indicates a substantial increase in invasion potential due to climate change. For invasive species, even a modest shift from negligible suitability (e.g., ~0) to low suitability (e.g., 0.1–0.2) can be ecologically transformative, as it opens previously inaccessible regions to potential colonization.

### 2.3. List of Alien Woody Species with High Invasion Risk in National Nature Reserves

Species in the top 10% with a distribution probability higher than the threshold for alien woody species in national nature reserves are defined as high-risk species. Under both current and future climate conditions, the potentially high-risk species include *Quercus robur* L., *Salix alba* L., *Populus tremuloides* Michx., *Acer negundo* L., *Sorbaria tomentosa* (Lindl.) Rehder, *Ribes nigrum* L., *Pseudotsuga menziesii* (Mirb.) Franco, *Betula pubescens* Ehrh., *Robinia pseudoacacia* L., *Populus balsamifera* L., among a total of 71 species (Table S3).

**Figure 5:**
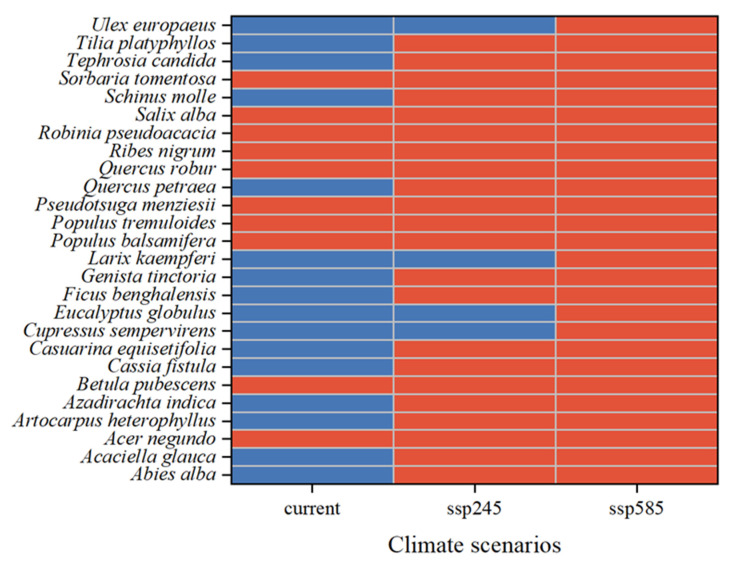
Alien woody plant species identified as high-risk in China’s National Nature Reserves under current and future climate scenarios (current, SSP245, SSP585). Red indicates species whose invasion probability in national nature reserves is at least 10% higher than the overall average across reserves. Blue indicates species with invasion probability in potential national parks ≤10% higher than in national nature reserves.

Species that pose a low risk under the current climate but are predicted to become high-risk in the future include *Ficus benghalensis* L., *Genista tinctoria* L., *Tephrosia candida* DC., *Quercus petraea* (Matt.) LieBlume, *Abies alba* Mill., *Tilia platyphyllos* Scop., *Casuarina equisetifolia* L., *Acaciella glauca* (L.) L. Rico, *Cassia fistula* L., *Schinus molle* L., *Artocarpus heterophyllus* Lam., and *Azadirachta indica* A. Juss. (Figure 5). These species are projected to emerge as high-risk invaders within nature reserves in the future.

## 3. Discussion

### 3.1. High-Risk Regions for AWP Invasion Under Climate Change Scenarios

Our study demonstrates that under current climate conditions, the highest risk of AWP invasion is concentrated in tropical and subtropical national nature reserves in southern China, such as Dongzhai Port (Hainan), Zhangjiangkou Mangrove (Guangdong), and Wuyishan (Fujian). These areas exhibit high mean invasion probabilities, primarily due to favorable temperature and precipitation conditions. However, under future climate scenarios—especially the high-emission SSP585 scenario—a marked shift occurs: invasion risk expands significantly toward northern and western reserves (Figure 2).

This shift in high-risk zones may largely driven by climate-mediated release from abiotic constraints. Historically, low temperatures have limited the northward and altitudinal expansion of most AWPs, many of which are native to tropical or subtropical climates [23]. With warming, however, the mean annual temperature (Bio1) is projected to increase in northern and western China [24,25], effectively raising the cold tolerance threshold for many invasive species [26]. In addition, changes in precipitation seasonality (Bio15) may create temporal resource pulses [27]—especially in arid western regions. These climatic shifts extend the growing season and improve seedling survival rates, enhancing the likelihood of long-term establishment.

Our findings align with previous studies, suggesting an increased risk of biological invasions in high-latitude regions in the context of global warming [28,29]. Similar patterns have been observed in the Eastern United States [11] and Mediterranean Europe [10]. However, China’s unique topography introduces regional complexity [30]. The rapid warming of the Tibetan Plateau creates novel ecological niches at high elevations [29,31]. Additionally, our multi-species approach—modeling 251 AWPs simultaneously—extends beyond prior work that focused on single species (e.g., *Robinia pseudoacacia*, *Quercus robur*), providing a broader landscape of emerging threats.

Given the climate-driven expansion of AWP distribution toward northern and western China, invasion prevention strategies must be regionally tailored and forward-looking. In southern reserves—such as Dongzhai Port (Hainan) and Wuyishan (Fujian)—which currently face high invasion pressure, management should emphasize the suppression of established seed sources and the prevention of secondary spread. This includes mechanical removal, habitat restoration, and the establishment of early detection and rapid response mechanisms to mitigate further ecological degradation [32]. In northern and western reserves—where risk is currently low but rapidly increasing—monitoring networks must be expanded to detect early signs of colonization, particularly during vulnerable phenological windows like the spring thaw [33]. In coastal reserves such as the Sanya Coral Reefs and Chongming Dongtan, the low probability of AWP invasion may be largely due to harsh abiotic conditions—such as high salinity, periodic inundation, and poor soil aeration—which are inherently unsuitable for the establishment and growth of most woody species [34,35]. These natural barriers, combined with effective habitat protection and quarantine regulations, help suppress potential invasions. While these coastal mechanisms are not directly transferable to inland ecosystems, their effectiveness underscores the value of targeted, context-specific management strategies for mitigating invasion risks under diverse ecological conditions [36]. Overall, adopting a spatially differentiated, climate-informed management framework is essential to safeguard biodiversity in China’s national nature reserves under future global change scenarios [13,20,37].

### 3.2. High-Risk AWP Species in China’s National Nature Reserves

Our multi-scenario analysis identified 71 AWP species as high-risk invaders in China’s national nature reserves under current and future climate conditions. Among these, several species consistently exhibited high distribution probabilities across all scenarios, including *Q. robur*, *S. tomentosa*, *S. alba*, *P. tremuloides*, *A. negundo*, and *R. pseudoacacia.* These species currently have high establishment potential in southern and eastern reserves and are projected to expand into northern and western regions with increasing climatic suitability. Additionally, 12 species previously considered low risk under the current climate—such as *F. benghalensis*, *S. molle*, *T. platyphyllos*, and *A. indica*—emerge as high-risk invaders under future climate scenarios (SSP245 and SSP585). Their distribution probabilities exceeded the average risk threshold of all reserves by more than 10%, indicating a significant invasion potential under warming conditions.

The success of these invasive species is closely related to their functional traits. Many high-risk alien woody plants (AWP) exhibit strong phenotypic plasticity, rapid growth rates, prolific reproductive capacity, and deep root systems. A representative example is *R. pseudoacacia* (black locust), native to temperate and subtropical North America. Introduced to Europe in the 17th century and to China in the 19th century, it has since been widely cultivated across Eurasia due to its fast growth, strong adaptability, and high economic value [38,39]. *R. pseudoacacia* can improve soil nutrient conditions through nitrogen fixation, creating a positive feedback mechanism that favors its own growth [40,41,42]. In the past 30–40 years, owing to the transformation of land use from arable land to artificial forests in the Tisza floodplain as well as poor forest management, the spread of *R. pseudoacacia* has been exceptionally rapid [43]. *P. tremuloides* and *S. alba* demonstrate strong competitive advantages in riparian zones and disturbed habitats through clonal reproduction and rapid canopy establishment [44]. Our study found that *Q. robur* has a high distribution probability under current and future climate scenarios. *Q. robur* is native to France and Italy in Europe. It has been introduced and cultivated in Xinjiang, Beijing and Shandong, China [45]. *Q. robur* can grow on many types of soil, including sandy, clay and limestone soils, giving it a wide distribution. Its resistance to wind, soot, short-term water and humidity, and the presence of dormant buds in its branches and strong tillering ability are also important reasons for its wide distribution.

Notably, several of the 12 species identified as emerging high-risk invaders—such as *A. indica*, *A. heterophyllus*, and *F. benghalensis*—are native to tropical or subtropical regions. These species are currently constrained by low-temperature thresholds, but under future climate scenarios, their suitable habitats are projected to shift toward higher latitudes and elevations. Climate warming reduces cold-related mortality, enhances seedling establishment, and allows thermophilic species to invade areas previously climatically inaccessible [46]. For instance, Osland and Feher (2020) demonstrated that warming winters reduce freeze events, enabling tropical species like *Schinus terebinthifolius* Raddi to expand into temperate zones [47]. Similarly, Keller (2021) found that elevated temperatures accelerate the life cycle of invasive plants, increasing survival and growth rates, which can lead to higher reproductive output [48]. These ecological and physiological responses to warming climates suggest that tropical invasive woody plants may increasingly establish and spread in temperate ecosystems, posing heightened invasion risks under future climate scenarios.

Effective management of high-risk AWP species necessitates differentiated strategies tailored to their ecological characteristics and invasion dynamics. For established invaders like *R. pseudoacacia* (black locust), which has been extensively planted for purposes such as ecological restoration and erosion control, management practices should focus on mitigating its invasive potential. Recommended measures include site-specific planting, avoiding monocultures, and integrating native species to promote biodiversity. In areas where *R. pseudoacacia* dominates, converting pure stands into mixed forests and implementing mechanical or biological control methods can help suppress its spread [49].

Beyond *R. pseudoacacia*, other high-risk AWP species identified in this study, such as *Q. robur*, *S. alba*, *P. tremuloides*, and *A. negundo*, require species-specific approaches based on their reproductive modes, dispersal vectors, and ecological niches [50]. For instance, clonal species like *S. alba* and *P. tremuloides* can rapidly colonize riparian zones and disturbed areas, necessitating the use of hydrological restoration or riparian buffer planting to limit establishment [51]. Wind-dispersed species such as *A. negundo* may require coordinated management at landscape scales, including seed source removal and the regulation of human-mediated dispersal pathways (e.g., roadside planting, horticulture) [52]. For species that establish dense canopies or alter soil properties (e.g., *Q. robur*), periodic understory monitoring and adaptive thinning practices may be employed to restore light availability and soil biota integrity [53]. Overall, integrated management frameworks that combine early detection, population containment, habitat restoration, and long-term monitoring are essential for curbing the expansion of high-risk AWP species, especially in climate-sensitive protected areas [7,54].

For emerging invasive species—many of which are currently undergoing climate-driven range expansions from tropical or subtropical regions—prevention and early detection are key [7,55,56]. A forward-looking strategy should involve the development of trait-based risk screening tools that evaluate attributes such as reproductive capacity, dispersal ability, and stress tolerance, which have been identified as reliable predictors of invasion success [57]. Moreover, strict regulation of horticultural introductions and commercial plant trade near the buffer zones of protected areas is needed to minimize propagule pressure [58]. Ecological restoration following invasive species removal should prioritize the reintroduction of native species with similar functional roles to prevent the emergence of vacant ecological niches and subsequent secondary invasions [59].

Our results also align with emerging international evidence that climate change is reshaping invasion risks in protected areas worldwide. In the eastern United States, climate warming of +2 °C is projected to drive invasive plant “hotspots” northeastward by ~200–250 km, exposing new reserves and natural areas to non-native species pressure [60]. In Europe, projections indicate that many alien deciduous tree species will expand their climatic niches northward or to higher elevations under 2061–2080 scenarios, while coniferous aliens may contract depending on local conditions [61]. In South America, case studies in Argentina show that woody invaders such as *Ligustrum lucidum* and *Acacia melanoxylon* are already threatening mountain forests, subtropical forests, and temperate grasslands, with risks amplified by climate change and land-use pressures [62]. These findings resonate with our China-wide analysis and reinforce that the methodological framework and adaptive management strategies we propose are transferable to other regions confronting climate-driven biological invasions.

This study is subject to several limitations. First, projections of invasion risk are inherently constrained by uncertainties in climate forecasts. While we utilized established GHG emission scenarios and GCM ensembles, unforeseen climatic shifts or deviations from these pathways could alter the projected distribution of suitable habitats for alien woody plants. Second, although the AUC statistic is widely used for model evaluation, its application over large geographic extents has recognized drawbacks. AUC values can be influenced by species prevalence and background sampling methods, potentially overestimating performance when applied across broad and heterogeneous landscapes. Third, the translation of continuous habitat suitability into binary presence-absence maps requires threshold selection, which introduces subjectivity. Different thresholding methods can produce substantially different range estimates, adding uncertainty to spatial projections of invasion risk. Future work would benefit from employing multi-model ensembles to capture climate uncertainty, using complementary evaluation metrics beyond AUC, and conducting sensitivity analyses on threshold selection.

## 4. Material and Methods

### 4.1. Species Data

All 251 AWPs were selected strictly from China’s National Invasive Species List, ensuring their documented impacts within Chinese ecosystems [63]. These AWP exhibit three defining attributes: rapid growth rates coupled with prolific reproductive potential, detrimental effects on native biodiversity and ecosystem functioning, and extensive geographical ranges associated with substantial invasive impacts [2,3,64]. The occurrence records of AWP were downloaded from the Global Biodiversity Information Facility (GBIF, https://www.gbif.org/ (accessed on 24 September 2025)). To capture the full global climatic niche of each species, we used all available global occurrence records for each species, without geographic filtering. Data for the 251 species were retrieved through multiple download tasks (see Data Availability Statement for a representative DOI). Taxonomic standardization was performed using the ‘Species Matching’ tool via the GBIF API, which aligns all names with the Catalog of Life backbone taxonomy. We accepted only records resolved to ‘ACCEPTED’ species names, automatically merging synonyms under their accepted names. We removed records of occurrences with the same latitude and longitude before inclusion in the modeling, and we corrected coordinates that did not match in latitude and longitude to minimize errors. We removed duplicate records within a given spatial resolution region to minimize the effect of sampling bias on the results.

### 4.2. Climatic and Topographic Data

We have selected climate change models from the global general circulation model, which are primarily used to project and assess future climate change trends and impacts [65]. We downloaded 19 bioclimatic variables (bio1–bio19) at 10 arc-minutes spatial resolution from the WorldClim database (https://www.worldclim.org/) to represent both current and future climate conditions [66]. The current climate dataset represents the baseline period of 1970–2000. For future climate projections (2081–2100), we selected three general circulation models (GCMs) under the Coupled Model Intercomparison Project Phase 6 (CMIP6): MIROC6, MPI-ESM1-2-HR, and MRI-ESM2-0. These models were chosen for their wide use and complementary simulation performances in East Asia. Two shared socioeconomic pathway scenarios (SSPs) were applied: SSP245, representing a moderate emission trajectory with partial mitigation efforts, and SSP585, representing a high-emission pathway with continued increases in greenhouse gas emissions. Under these scenarios, global average temperature increases are projected to remain within 2 °C for SSP245 and potentially exceed 4 °C for SSP585 by the end of the century. For each of the three GCMs and two future scenarios, we extracted four key variables (bio1, bio4, bio12, and bio15) and calculated their weighted average to generate ensemble climate layers.

The selection of these four variables—mean annual temperature (bio1), temperature seasonality (bio4), annual precipitation (bio12), and precipitation seasonality (bio15)—was based on two criteria: (1) ecological relevance for plant growth and invasion dynamics, and (2) low multicollinearity (|r| < 0.8) among variables, assessed via Pearson correlation analysis using IBM SPSS Statistics (version 26). This ensured that the final model inputs captured climatic gradients critical to the potential distribution of AWPs, while minimizing redundancy. All environmental layers were spatially standardized and resampled to 10 km resolution using ArcGIS 10.8 to ensure consistency in modeling.

We used five terrain data factors from https://www.earthenv.org, which include elevation, roughness, terrain roughness index (TRI), vector roughness measure (VRM), and terrain position index (TPI), all at 10 km resolution [67].

### 4.3. National Nature Reserve Data

The spatial boundaries and administrative attributes of China’s national nature reserves were obtained from the National Ecological Protection Database, managed by the Ministry of Ecology and Environment. The dataset includes 479 reserves designated before 2020, covering all major ecosystem types (e.g., forests, wetlands, grasslands) and endangered species habitats (e.g., giant pandas, Siberian tigers). The map of China’s nature reserves was created using the Resource and Environmental Science and Data Center (https://www.resdc.cn/Default.aspx (accessed on 24 September 2025)) and World Database on Protected Areas (https://www.protectedplanet.net/).

### 4.4. Species Distribution Modeling

Species distribution modeling is a tool used to predict the potential distribution of species under different environmental conditions [68]. These models are often based on correlations between species and environmental factors, such as climate, topography, and soils. Using species occurrence data and environmental data, species distribution models can be constructed to simulate the distribution of species under current and future climatic conditions. MaxEnt models sometimes outperform other techniques in predicting the potential area of a species, requiring only a small sample size of presence-only data [69,70].

The distribution of 251 AWP was predicted using MaxEnt, which predicts the probability and suitability of the distribution of AWP using only presence-only species distribution data and environmental variables [71]. The results of the MaxEnt model yielded species distribution probabilities ranging from 0 to 1. We selected a fixed regularization multiplier (RM) value of 2.0 across all species based on preliminary sensitivity analyses. For a representative subset of species, we tested RM values ranging from 0.5 to 5.0 and evaluated model performance. We found that RM = 2.0 consistently yielded optimal or near-optimal performance for the majority of species, while effectively reducing overfitting—particularly for species with sparse or spatially clustered occurrence records. To ensure methodological consistency and facilitate cross-species comparisons, we applied this value uniformly across all models. Both the randomized training test data and test data for each dataset were repeated for four runs, with the regularization multiplier (beta) was 2.0 to produce a smooth and general response curve that represents the actual biological behavior and the other parameters at default values [72]. We used the logistic output format transformation to estimate the plant species distribution probability, which ranges from 0 (lowest) to 1 (highest) [73].

The area under the ROC Curve (AUC) value of the MaxEnt model can be used to evaluate the model’s performance and prediction accuracy [70]. The AUC value ranges from 0 to 1, where 0.5 indicates that the model’s prediction is equivalent to a random guess and 1 indicates that the model’s prediction is completely accurate [74]. In general, the closer the AUC value is to 1, the better the performance of the model. In this study, we considered AUC values greater than 0.7 as good model performance and discarded those with AUC values less than 0.7 [75].

### 4.5. Zonation Spatial Prioritization Analysis

To identify priority areas for managing AWP under climate change, we employed Zonation GUI 4.0.0 for spatial conservation planning [76]. The framework integrates species distribution models (MaxEnt outputs) to generate hierarchical priority layers through iterative removal of grids with the lowest conservation value, emphasizing connectivity and multi-species risk aggregation [69,77]. Current and future climate scenario (SSP245 and SSP585) distribution probability layers (.asc format) for 251 AWP were imported at 10 km resolution, with the “core-area” algorithm applied to prioritize high-risk invasion hotspots [78]. Zonation generated spatial priority layers for both climate scenarios, with values approaching 1 indicating regions requiring urgent management due to overlapping multi-species invasion risks [76].

To evaluate climate-driven shifts in risk, provincial-level monitoring priorities were derived by overlaying Zonation outputs with national nature reserve boundaries in ArcGIS 10.8. Mean priority scores were extracted for each reserve using Zonal Statistics, and provincial averages were calculated via Summary Statistics based on administrative attributes.

In order to assess the impacts of climate change on the risk of invasion, the logarithmic response ratio (RR) was calculated as RR = ln (Xf/Xc), where Xc and Xf represent the mean invasion risk scores for grid cells within each nature reserve under current and future climate conditions, respectively [79]. This metric is employed to quantify the proportional change in invasion risk, with positive RR values denoting elevated future risk and negative values indicating reduced vulnerability. The RR transformation provides a standardized measure of climate-mediated risk shifts across reserves while maintaining the ratio’s original proportionality [80].

### 4.6. Statistical Analysis

To investigate the influence of climate change on woody plant invasions in protected areas, we conducted a regional statistical analysis using the information on protected areas and the potential distribution areas of AWP under different climate scenarios [28]. The probability of distribution of AWP in protected areas under the current climate scenario was compared with that under the future climate scenario [11]. If the distribution of AWP under the future climate scenario is higher than the current one, then climate change can exacerbate woody plant invasion in protected areas. If the distribution of AWP under the future climate scenario is lower than the current one, then climate change cannot exacerbate woody plant invasion in protected areas.

Finally, we used the average distribution of alien woody species in national nature reserves to calculate the distribution probability of each of the 251 invasive woody species in the nature reserves under different climate scenarios. Species were considered high-risk if their mean probability of occurrence in a potential protected area exceeded the average across national nature reserves by more than 10% [22]. Similar threshold-based classification approaches have been widely applied in invasion risk studies [81,82,83].

## 5. Conclusions

Understanding spatial–temporal invasion risks of alien woody plants (AWPs) under climate change is vital for China’s nature reserves. Modeling 251 AWPs across 479 reserves revealed current invasion hotspots in the south, with future risks shifting north and west. Seventy-one high-risk species were identified, including *Q. robur*, *S. alba*, and *R. pseudoacacia*, while others like *F. benghalensis* are expected to spread further amid global change. Regional, climate-adaptive management is essential: southern reserves should prioritize containment, northern and western areas focus on early detection and monitoring. This study provides a practical framework for biodiversity conservation amid global change.

## Data Availability

Occurrence data for the 251 alien woody plants were obtained from the Global Biodiversity Information Facility (GBIF) through multiple download tasks conducted between 30 June and 2 July 2023. The data are publicly available through the following DOIs: https://doi.org/10.15468/dl.fyy9xv, https://doi.org/10.15468/dl.sayetr, … (and so on for all DOIs). The complete list of GBIF dataset citations is provided as Supplementary Material S1. Climatic data were sourced from WorldClim (https://www.worldclim.org), and topographic data from the EarthEnv project (https://www.earthenv.org).

## Supplementary Materials

The following supporting information can be downloaded at: https://www.mdpi.com/article/10.3390/plants14193006/s1, Table S1: Training AUC and test AUC for 251 Alien Woody Plants; Table S2: Logarithmic response ratios (RR) for national nature reserves under the ssp245 and ssp585 climate scenarios; Table S3: Alien woody plant species identified as high-risk (≥10% above the mean invasion probability) in China’s national nature reserves under current and future climate scenarios (Current, SSP245, SSP585); Supplementary Material S1: GBIF Data Citations.
