# Supplementary material for: Risk Assessment of Alien Woody Plants in China’s National Nature Reserves Under Climate Change
Source: Plants (Basel). 2025 Sep 28;14(19):3006. doi: 10.3390/plants14193006 (PMC12526335; doi:10.3390/plants14193006)
Supplement: Supplementary file 1 [file plants-14-03006-s001.zip › plants-3877665-supplementary.pdf]

Table S1 Training AUC and test AUC for 251 Alien Woody Plants

Species	Training AUC	Test AUC	Species	Training AUC	Test AUC
Cryptomeria japonica	0.9137	0.9124	*Ficus rubiginosa*	0.9503	0.9486
Chamaecyparis lawsoniana	0.89	0.8898	*Cecropia peltata*	0.9326	0.9308
Cupressus sempervirens	0.8967	0.8959	*Fagus sylvatica*	0.7571	0.7569
Juniperus occidentalis	0.9671	0.9659	*Castanea crenata*	0.9586	0.9582
Juniperus virginiana	0.8367	0.8366	*Castanea sativa*	0.8076	0.8075
Cedrus deodara	0.9131	0.9123	*Quercus cerris*	0.9042	0.9039
Abies alba	0.844	0.8438	*Quercus coccifera*	0.9136	0.9131
Abies amabilis	0.9752	0.9744	*Quercus petraea*	0.8144	0.8142
Abies grandis	0.9062	0.9058	*Quercus robur*	0.9504	0.951
Abies nordmanniana	0.9475	0.9469	*Quercus rubra*	0.7724	0.7722
Abies procera	0.96	0.9593	*Myrica gale*	0.8062	0.8061
Abies sachalinensis	0.9802	0.9779	*Juglans ailanthifolia*	0.9713	0.9702
Pseudotsuga menziesii	0.7883	0.7881	*Juglans cinerea*	0.9357	0.9355
Larix decidua	0.8151	0.815	*Juglans nigra*	0.8442	0.8439
Larix kaempferi	0.9098	0.9093	*Casuarina cunninghamiana*	0.922	0.9204
Picea abies	0.708	0.7077	*Casuarina equisetifolia*	0.8951	0.8937
Picea sitchensis	0.887	0.8865	*Casuarina glauca*	0.982	0.9756
Pinus banksiana	0.9052	0.9042	*Allocasuarina littoralis*	0.9489	0.9482
Pinus canariensis	0.9703	0.9665	*Alnus glutinosa*	0.7148	0.7146
Pinus caribaea	0.9592	0.9547	*Alnus rubra*	0.9607	0.9599
Pinus contorta	0.851	0.8505	*Betula pubescens*	0.7016	0.7013
Pinus elliottii	0.9677	0.9666	*Carpinus betulus*	0.7933	0.7932
Pinus glabra	0.9869	0.986	*Averrhoa carambola*	0.9133	0.9103

Pinus halepensis	0.9051	0.9048	*Pentadesma butyracea*	0.9761	0.9731
Pinus luchuensis	0.9872	0.9841	*Mammea americana*	0.9533	0.9443
Pinus monticola	0.9589	0.9577	*Galphimia glauca*	0.9546	0.9512
Pinus nigra	0.841	0.8407	*Dovyalis caffra*	0.9684	0.9671
Pinus oocarpa	0.966	0.9639	*Populus balsamifera*	0.8276	0.8269
Pinus parviflora	0.9772	0.9761	*Populus deltoides*	0.8425	0.8419
Pinus patula	0.9582	0.9566	*Populus nigra*	0.7775	0.7772
Pinus pinaster	0.888	0.8877	*Populus tremuloides*	0.8011	0.8004
Pinus pinea	0.927	0.9264	*Salix alba*	0.976	0.9559
Pinus ponderosa	0.8974	0.8967	*Salix nigra*	0.8941	0.8934
Pinus radiata	0.923	0.9225	*Salix repens*	0.8219	0.8216
Pinus rigida	0.9517	0.9513	*Hevea brasiliensis*	0.9303	0.9264
Pinus strobus	0.8081	0.8079	*Aleurites moluccanus*	0.9359	0.9336
Pinus taeda	0.9401	0.9398	*Reutealis trisperma*	0.9652	0.9369
Pinus thunbergii	0.9657	0.965	*Garcia nutans*	0.9661	0.9465
Annona squamosa	0.8982	0.8957	*Hura crepitans*	0.933	0.9304
Persea americana	0.8654	0.8636	*Melianthus major*	0.9751	0.9742
Cinnamomum verum	0.9267	0.922	*Sonneratia apetala*	0.9907	0.988
Arenga pinnata	0.9528	0.9417	*Callistemon linearis*	0.9762	0.9716
Borassus flabellifer	0.9667	0.9622	*Callistemon rigidus*	0.9435	0.9219
Washingtonia filifera	0.9398	0.9361	*Callistemon viminalis*	0.9289	0.927
Washingtonia robusta	0.9319	0.9275	*Melaleuca hypericifolia*	0.9868	0.9824
Phytelephas macrocarpa	0.974	0.9692	*Corymbia citriodora*	0.9445	0.9425
Roystonea oleracea	0.9688	0.9649	*Corymbia maculata*	0.9726	0.9711
Elaeis guineensis	0.9364	0.935	*Corymbia torelliana*	0.9645	0.9598
Archontophoenix cunninghamiana	0.9792	0.9766	*Eucalyptus benthamii*	0.9782	0.9723

Ptychosperma elegans	0.9749	0.9672	*Eucalyptus botryoides*	0.9791	0.9782
Ptychosperma macarthurii	0.9534	0.9486	*Eucalyptus brassiana*	0.9821	0.9742
Berberis darwinii	0.9584	0.9578	*Eucalyptus camaldulensis*	0.8687	0.8673
Berberis microphylla	0.9814	0.9804	*Eucalyptus cinerea*	0.9661	0.9638
Berberis thunbergii	0.8005	0.8004	*Eucalyptus cladocalyx*	0.9762	0.9752
Berberis vulgaris	0.8016	0.8014	*Eucalyptus cloeziana*	0.9897	0.9882
Hakea salicifolia	0.9765	0.9757	*Eucalyptus diversicolor*	0.993	0.9909
Grevillea banksii	0.9692	0.9655	*Eucalyptus globulus*	0.9038	0.9028
Grevillea robusta	0.8797	0.8783	*Eucalyptus gomphocephala*	0.9849	0.9785
Dillenia suffruticosa	0.9819	0.98	*Eucalyptus grandis*	0.9741	0.9669
Ribes alpinum	0.8234	0.8233	*Eucalyptus gunnii*	0.9826	0.9788
Ribes aureum	0.8273	0.8266	*Eucalyptus lehmannii*	0.9938	0.9932
Ribes multiflorum	0.9802	0.9581	*Eucalyptus megacornuta*	0.985	0.9717
Ribes nigrum	0.7526	0.7523	*Eucalyptus nitens*	0.9921	0.9881
Ribes uva-crispa	0.7693	0.7691	*Eucalyptus pellita*	0.9633	0.9534
Copaifera officinalis	0.9621	0.9427	*Eucalyptus robusta*	0.9508	0.9476
Tamarindus indica	0.8629	0.8621	*Eucalyptus saligna*	0.9736	0.9726
Cassia fistula	0.8871	0.8838	*Eucalyptus sideroxylon*	0.9653	0.9644
Senna multijuga	0.9312	0.9284	*Eucalyptus tereticornis*	0.9292	0.9283
Senna siamea	0.8883	0.8871	*Eucalyptus urophylla*	0.968	0.9559
Senna spectabilis	0.9109	0.9096	*Eucalyptus viminalis*	0.9454	0.9446
Schizolobium parahyba	0.942	0.9379	*Metrosideros excelsa*	0.9815	0.9808
Delonix regia	0.8536	0.8521	*Syzygium malaccense*	0.9423	0.9401
Mimosa pigra	0.8516	0.8501	*Syzygium paniculatum*	0.9752	0.9731
Acaciella glauca	0.9377	0.9239	*Psidium guineense*	0.9098	0.9079
Albizia lebbeck	0.8831	0.8811	*Pimenta dioica*	0.9502	0.9451

Acacia adunca	0.9954	0.9931	*Heterotis rotundifolia*	0.9488	0.9462
Acacia decurrens	0.9578	0.9567	*Staphylea pinnata*	0.9264	0.926
Acacia elata	0.9877	0.9872	*Schinus molle*	0.8828	0.8823
Acacia fimbriata	0.9798	0.9788	*Acer negundo*	0.7071	0.707
Acacia holosericea	0.9492	0.9484	*Acer platanoides*	0.7225	0.7223
Acacia implexa	0.9516	0.9512	*Acer pseudoplatanus*	0.7535	0.7535
Acacia iteaphylla	0.9829	0.9822	*Aesculus hippocastanum*	0.7683	0.7682
Acacia mangium	0.9433	0.9397	*Melicoccus bijugatus*	0.9548	0.9525
Acacia melanoxylon	0.8971	0.8967	*Cedrela odorata*	0.8879	0.8858
Acacia paradoxa	0.9561	0.9558	*Azadirachta indica*	0.8586	0.8572
Acacia podalyriifolia	0.9665	0.9649	*Triumfetta bogotensis*	0.9542	0.9485
Myroxylon balsamum	0.9411	0.9339	*Tilia cordata*	0.7619	0.7615
Styphnolobium japonicum	0.9334	0.9308	*Tilia platyphyllos*	0.8283	0.828
Cytisus nigricans	0.9597	0.9591	*Sterculia apetala*	0.9404	0.9372
Cytisus scoparius	0.7676	0.7673	*Pachira aquatica*	0.9343	0.9315
Chamaecytisus ratisbonensis	0.9849	0.983	*Cochlospermum religiosum*	0.9682	0.9502
Spartium junceum	0.8779	0.8774	*Helianthemum apenninum*	0.9224	0.9218
Genista germanica	0.9278	0.9275	*Helianthemum ledifolium*	0.9545	0.9529
Genista pilosa	0.9012	0.901	*Triplaris americana*	0.944	0.9412
Genista sagittalis	0.9287	0.9285	*Triplaris cumingiana*	0.9623	0.9563
Genista tinctoria	0.8121	0.8118	*Atriplex nummularia*	0.9496	0.9488
Ulex europaeus	0.8272	0.827	*Phytolacca dioica*	0.9392	0.9381
Amorpha fruticosa	0.8203	0.8197	*Philadelphus coronarius*	0.7989	0.7987
Dalbergia sissoo	0.9257	0.9196	*Cornus sanguinea*	0.7714	0.7714
Tipuana tipu	0.9519	0.9498	*Manilkara zapota*	0.9208	0.9193
Tephrosia candida	0.9608	0.9517	*Daboecia cantabrica*	0.9839	0.9829

Erythrina poeppigiana	0.9561	0.9534	*Calluna vulgaris*	0.7062	0.7061
Hippocrepis emerus	0.9118	0.9115	*Rhododendron ferrugineum*	0.9536	0.953
Gliricidia sepium	0.9036	0.9019	*Rhododendron hirsutum*	0.9765	0.9758
Robinia pseudoacacia	0.7225	0.7222	*Rhododendron ponticum*	0.9096	0.9091
Medicago arborea	0.9648	0.9638	*Cinchona pubescens*	0.9677	0.9659
Polygala myrtifolia	0.9389	0.9385	*Voacanga africana*	0.9621	0.9545
Rubus moluccanus	0.9478	0.9462	*Gomphocarpus fruticosus*	0.9114	0.9102
Rosa pendulina	0.922	0.9214	*Cordia alliodora*	0.909	0.9066
Physocarpus opulifolius	0.8454	0.845	*Solanum anguivi*	0.9128	0.91
Prunus domestica	0.7961	0.796	*Ligustrum vulgare*	0.7574	0.7573
Sorbaria tomentosa	0.9774	0.9706	*Fraxinus americana*	0.8943	0.8937
Spiraea alba	0.8649	0.8645	*Fraxinus angustifolia*	0.87	0.8698
Spiraea douglasii	0.9018	0.9011	*Fraxinus excelsior*	0.7464	0.7462
Pyracantha coccinea	0.8484	0.8482	*Fraxinus pennsylvanica*	0.8417	0.8412
Amelanchier lamarckii	0.9466	0.9462	*Fraxinus uhdei*	0.9579	0.9526
Amelanchier ovalis	0.9034	0.9031	*Jacaranda mimosifolia*	0.8864	0.8848
Cotoneaster simonsii	0.9575	0.9572	*Catalpa speciosa*	0.933	0.9321
Rhamnus alaternus	0.8932	0.8929	*Tabebuia rosea*	0.9122	0.91
Ulmus americana	0.8733	0.8729	*Spathodea campanulata*	0.8909	0.8888
Ulmus laevis	0.8664	0.8661	*Lantana camara*	0.7383	0.7376
Ulmus minor	0.8022	0.8021	*Tectona grandis*	0.9023	0.9001
Artocarpus altilis	0.9231	0.9222	*Lonicera xylosteum*	0.7574	0.7571
Artocarpus heterophyllus	0.8996	0.897	*Symphoricarpos orbiculatus*	0.9193	0.9188
Ficus benghalensis	0.9224	0.92	*Billardiera heterophylla*	0.9753	0.9747
Ficus religiosa	0.9155	0.9121			

Table S2 Logarithmic response ratios (RR) for national nature reserves under the ssp245 and ssp585 climate scenarios

English name	RR245	RR585	Province
Zibaishan national nature reserve	0.003845	-0.018032	Hubei Province
Qomolangma national nature reserve	0.141658	0.300361	Tibet Autonomous Region
Zhouzhi national nature reserve	0.072163	0.080108	Shaanxi Province
Zhongyangzhan Black-billed Capercaillienational nature reserve	0.308133	0.420129	Heilongjiang Province
Zhenbaodao Wetland national nature reserve	0.128187	0.17204	Sichuan Province
Changsha Gongmanational nature reserve	0.048299	0.225681	Hunan Province
Changning Zhuhai national nature reserve	-0.015013	-0.104965	Guangdong Province
Yangtze River Xinluo Section Baiji Dolphin national nature reserve	-0.042517	-0.032557	Yunnan Province
Upper Yangtze Rare and Endemic Fish national nature reserve	-0.022459	-0.076269	Hainan Province
Zhalong national nature reserve	0.040955	0.101566	Guangxi Zhuang Autonomous Region
Dawei Shan national nature reserve	-0.008588	-0.014705	Shanxi Province
Yunkai Shan national nature reserve	0.006955	-0.001319	Anhui Province
Yuanjiang national nature reserve	-0.000571	0.013051	Jilin Province
Youhao Wetlands national nature reserve	0.184096	0.338185	Gansu Province
Yongde Daxueshan national nature reserve	0.043782	0.05991	Henan Province
Yingzuijie national nature reserve	-0.063385	-0.117257	Hunan Province
Yingge Ling national nature reserve	-0.005257	-0.026896	Hainan Province
Yinzhulaoshan national nature reserve	-0.039327	-0.078071	Guangxi Zhuang Autonomous Region
Yintiaoling national nature reserve	0.014884	-0.008232	Chongqing Municipality
Yiwulü Shan national nature reserve	0.029796	-0.014964	Liaoning Province
Yaoshan national nature reserve	0.08841	0.109056	Yunnan Province
Yangming Shan national nature reserve	-0.045304	-0.107611	Hunan Province
Yangcheng Manghe Macaque national nature reserve	-0.004158	0.000181	Shanxi Province

Chinese alligator national nature reserve	-0.033573	-0.017709	Anhui Province
Yanminghu national nature reserve	0.059096	0.090843	Jilin Province
Yanchiwan national nature reserve	-0.117205	-0.229718	Gansu Province
Taoyuandong national nature reserve	-0.033948	-0.069471	Hunan Province
Yanan Huanglongshan Hemaji national nature reserve	0.037333	0.048979	Shaanxi Province
Yading national nature reserve	0.148076	0.263779	Sichuan Province
Brahmaputra Jiang Zhongyouhegu Heijinghe national nature reserve	0.165778	0.330731	Tibet Autonomous Region
Yarlung Zangbo Daxiagu national nature reserve	0.131199	0.195181	Tibet Autonomous Region
Xuebao Shan national nature reserve	-0.00735	-0.044441	Chongqing Municipality
Xuebaoding national nature reserve	0.158991	0.251073	Sichuan Province
Xinglong Shan national nature reserve	0.092212	0.135746	Gansu Province
Xingkai Hu national nature reserve	0.105807	0.120947	Heilongjiang Province
Xingdou Shan national nature reserve	-0.033598	-0.083534	Hubei Province
Xinxiang Yellow River Wetland Birds national nature reserve	-0.024421	-0.098088	Henan Province
Xinqing Baitouhe national nature reserve	0.085485	0.244661	Heilongjiang Province
Xiaozhaizigou national nature reserve	0.11869	0.148301	Sichuan Province
Xiaoxi national nature reserve	-0.060811	-0.111369	Hunan Province
Xiaowutai Shan national nature reserve	0.043477	0.119087	Hebei Province
Xiaoqinling national nature reserve	0.017088	-0.005075	Henan Province
Xiaojinsiguniang Shan national nature reserve	0.149792	0.315865	Sichuan Province
Xianghai national nature reserve	-0.005507	0.032663	Jilin Province
Xilinhe national nature reserve	0.116669	0.248773	Heilongjiang Province
Xishui Central Subtropical Evergreen Broad-leaved Forest national nature reserve	-0.031491	-0.114124	Guizhou Province
Xilingol Grassland national nature reserve	0.17352	0.145556	Inner Mongolia Autonomous Region
Xitianshan national nature reserve	-0.055941	0.030524	Xinjiang Uyghur Autonomous Region
Xishuangbanna national nature reserve	-0.00167	-0.009178	Yunnan Province

Western Ordos national nature reserve	-0.085558	-0.227402	Inner Mongolia Autonomous Region
Xidongting Lake national nature reserve	-0.025305	-0.01339	Hunan Province
Wuzhishan national nature reserve	0.001386	-0.014621	Hainan Province
Wulushan national nature reserve	0.03185	0.043738	Shanxi Province
Wufeng Houhe national nature reserve	-0.025864	-0.060919	Hubei Province
Wudalianchi national nature reserve	0.224967	0.320972	Heilongjiang Province
Wuyunjie national nature reserve	-0.068609	-0.11026	Hunan Province
Wuyuer River national nature reserve	0.10871	0.161553	Heilongjiang Province
Wui Ling national nature reserve	0.181607	0.33377	Heilongjiang Province
Wuyanling national nature reserve	-0.048157	-0.08767	Zhejiang Province
Wumeng Shan national nature reserve	-0.025478	-0.061093	Yunnan Province
Wuma River Sable national nature reserve	0.085401	0.255022	Heilongjiang Province
Wulanba national nature reserve	0.148066	0.215097	Inner Mongolia Autonomous Region
Mongolian wild ass national nature reserve	-0.173112	-0.33311	Inner Mongolia Autonomous Region
Wolong national nature reserve	0.122796	0.208578	Sichuan Province
Wenshan national nature reserve	0.020042	0.025061	Yunnan Province
Weichang Hongsongwa national nature reserve	0.038506	0.159362	Hebei Province
Wanglang national nature reserve	0.161037	0.294789	Sichuan Province
Wangqing national nature reserve	0.097336	0.182117	Jilin Province
Tömür Peak national nature reserve	-0.092472	-0.075742	Xinjiang Uyghur Autonomous Region
Tumuji national nature reserve	0.014377	0.058349	Inner Mongolia Autonomous Region
Tongling Freshwater Dolphin national nature reserve	-0.031704	-0.009504	Anhui Province
Tongbo Shan national nature reserve	-0.04051	-0.089034	Jiangxi Province
Tianhua Shan national nature reserve	0.029672	0.015301	Shaanxi Province
Tianfuzhi Shan national nature reserve	0.097748	0.175354	Jilin Province
Taohongling Elk national nature reserve	-0.039051	-0.066911	Jiangxi Province

Tao River national nature reserve	0.146322	0.296993	Gansu Province
Tangjia River national nature reserve	0.045541	0.041464	Sichuan Province
Taizi Shan national nature reserve	0.127545	0.239272	Gansu Province
Taihangshan Macaque national nature reserve	-0.003348	-0.008109	Henan Province
Taiping Gou national nature reserve	0.147479	0.240213	Heilongjiang Province
Taiwen River national nature reserve	-0.001381	-0.014098	Shanxi Province
Taibai Shan national nature reserve	0.044926	0.055894	Shaanxi Province
Tarim Populus euphratica national nature reserve	-0.096652	-0.131549	Xinjiang Uyghur Autonomous Region
Songhua River Three Lakes national nature reserve	0.103339	0.136788	Jilin Province
Sihong Hongze Lake Wetland national nature reserve	-0.045216	-0.078102	Jiangsu Province
Shuangtaihekou national nature reserve	0.00243	-0.016877	Liaoning Province
Shimentai national nature reserve	-0.017533	-0.048372	Guangdong Province
Shiwangdasha national nature reserve	-0.01795	-0.056628	Guangxi Zhuang Autonomous Region
Shengshan national nature reserve	0.24556	0.385771	Heilongjiang Province
Shengjin Lake national nature reserve	-0.018561	0.008498	Anhui Province
Shenlong Jia national nature reserve	0.019579	0.006772	Hubei Province
Shaanxi Ziwuling national nature reserve	0.044484	0.04572	Shaanxi Province
Shaanxi Motianling national nature reserve	0.005303	-0.016181	Shaanxi Province
Shaanxi Micang Shan national nature reserve	-0.008276	-0.052983	Shaanxi Province
Shaanxi Heihe Rare Aquatic Animals national nature reserve	0.042387	0.031955	Shaanxi Province
Shaanxi Danfeng Wuguanhe Rare Aquatic Animals national nature reserve	-0.023745	-0.073718	Shaanxi Province
Selin Co national nature reserve	0.1646	0.377199	Tibet Autonomous Region
Sangyuan national nature reserve	0.040887	0.031101	Shaanxi Province
Sanjiangyuan national nature reserve	0.019279	0.135473	Qinghai Province
Sanjiang national nature reserve	0.156343	0.214241	Heilongjiang Province
Saihanwula national nature reserve	0.157735	0.231076	Inner Mongolia Autonomous Region

Ruoergai Wetland national nature reserve	0.14793	0.31678	Sichuan Province
Raohe Northeast Black Bee national nature reserve	0.124898	0.175695	Heilongjiang Province
Qinghai Lake national nature reserve	0.062378	0.210222	Qinghai Province
Qiangtang national nature reserve	-0.106553	-0.135491	Tibet Autonomous Region
Qianjiadong national nature reserve	-0.047574	-0.109467	Guangxi Zhuang Autonomous Region
Qi Si Zimei Shan national nature reserve	-0.021813	-0.053573	Hubei Province
Qixinglazi Northeast Tiger national nature reserve	0.145313	0.203237	Heilongjiang Province
Poyang Lake Nanji Wetland national nature reserve	-0.028653	-0.011296	Jiangxi Province
Poyang Lake Migratory Birds national nature reserve	-0.028613	-0.011458	Jiangxi Province
Pinghe Liang national nature reserve	0.038033	0.041126	Shaanxi Province
Panzhong national nature reserve	0.259787	0.399828	Heilongjiang Province
Longgang national nature reserve	-0.060985	-0.129797	Guangxi Zhuang Autonomous Region
Ningxia Luoshan national nature reserve	-0.006792	-0.07773	Ningxia Hui Autonomous Region
Ningxia Helanshan national nature reserve	-0.048631	-0.081733	Ningxia Hui Autonomous Region
Inner Mongolia Helanshan national nature reserve	0.009389	0.096571	Inner Mongolia Autonomous Region
Inner Mongolia Daqingshan national nature reserve	0.023916	0.03504	Inner Mongolia Autonomous Region
Inner Mongolia Daheshan national nature reserve	0.013911	-0.015365	Inner Mongolia Autonomous Region
Naoili River national nature reserve	0.117205	0.133879	Heilongjiang Province
Nanyue Hengshan national nature reserve	-0.052286	-0.08094	Hunan Province
Nanyang Dinosaur Eggs Fossil Group national nature reserve	-0.080135	-0.145668	Henan Province
Nanweng River national nature reserve	0.294264	0.395367	Heilongjiang Province
Namocu Wetland national nature reserve	0.14816	0.310432	Sichuan Province
Nanling national nature reserve	-0.034661	-0.070864	Guangdong Province
Nanhua Shan national nature reserve	0.098196	0.133388	Ningxia Hui Autonomous Region
Nangun River national nature reserve	0.013163	0.020882	Yunnan Province
Meigu Dawengding national nature reserve	0.079958	0.111585	Heilongjiang Province

Meihuashan national nature reserve	-0.023575	-0.058267	Hubei Province
Maolan national nature reserve	0.078019	0.096932	Heilongjiang Province
Maolangou national nature reserve	0.016723	0.070002	Jilin Province
Maojingba national nature reserve	0.056774	0.137897	Heilongjiang Province
Mangkang Yunnan Golden Snub-nosed Monkey national nature reserve	-0.139285	-0.387624	Gansu Province
Maidika Wetland national nature reserve	-0.005126	-0.035568	Sichuan Province
Mapangyongco Wetland national nature reserve	0.057871	0.087875	Sichuan Province
Mabian Dawengding national nature reserve	-0.016838	-0.034658	Fujian Province
Lop Nur Wild Camel national nature reserve	-0.061641	-0.116916	Guizhou Province
Luan River Upper Reaches national nature reserve	0.092194	0.244249	Heilongjiang Province
Longbao national nature reserve	0.012745	-0.0091	Hebei Province
Longxi-Hongkou national nature reserve	0.160809	0.281378	Tibet Autonomous Region
Longgan Lake national nature reserve	0.102047	0.297306	Tibet Autonomous Region
Liupan Shan national nature reserve	0.150067	0.289335	Tibet Autonomous Region
Liubuxi national nature reserve	0.06439	0.083331	Sichuan Province
Lingfeng national nature reserve	-0.1429	-0.183071	Xinjiang Uyghur Autonomous Region
Lingwu Baiji Tan national nature reserve	0.030226	0.151929	Hubei Province
Junzifeng national nature reserve	0.156166	0.327572	Qinghai Province
科尔沁 national nature reserve	0.08031	0.091179	Sichuan Province
Kunyu Shan national nature reserve	-0.026954	-0.017585	Hubei Province
Kuankeshui national nature reserve	0.109676	0.170567	Ningxia Hui Autonomous Region
Kekexili national nature reserve	-0.071697	-0.113192	Hunan Province
Micang Shan national nature reserve	0.01711	0.248529	Heilongjiang Province
Jianfeng Ling national nature reserve	-0.080918	-0.181354	Ningxia Hui Autonomous Region
Leiwuzhi Elk national nature reserve	0.050022	0.084847	Shanxi Province
Laoyeling Northeast Tiger national nature reserve	0.017331	-0.013588	Liaoning Province

Jiuzhaigou national nature reserve	0.091578	0.136938	Gansu Province
Jiuyishan national nature reserve	0.069434	0.092829	Sichuan Province
Jiwan Shan national nature reserve	0.066139	0.069355	Shanxi Province
Jiulian Shan national nature reserve	0.153351	0.315469	Tibet Autonomous Region
Jinzhongshan Black-necked Long-tailed Partridge national nature reserve	-0.024951	-0.058498	Guizhou Province
Jinzhai Tianma national nature reserve	0.1023	0.172342	Heilongjiang Province
Jiaozi Shan national nature reserve	0.080913	0.107864	Shaanxi Province
Jiangle Longqishan national nature reserve	0.083258	0.26489	Heilongjiang Province
Jiangxi Matoushan national nature reserve	-0.008909	-0.028189	Shandong Province
Kuankeshui national nature reserve	-0.023449	-0.081966	Guizhou Province
Kekexili national nature reserve	-0.121102	-0.189159	Qinghai Province
Keerqin national nature reserve	-0.005848	0.02606	Inner Mongolia Autonomous Region
Junzifeng national nature reserve	-0.038478	-0.060315	Fujian Province
Jiuzhaigou national nature reserve	0.171498	0.320536	Sichuan Province
Jiuyishan national nature reserve	-0.048675	-0.124822	Hunan Province
Jiwan Shan national nature reserve	-0.063483	-0.113627	Guangxi Zhuang Autonomous Region
Jiulian Shan national nature reserve	-0.042668	-0.08206	Jiangxi Province
Jinzhongshan Black-necked Long-tailed Partridge national nature reserve	-0.032963	-0.052619	Guangxi Zhuang Autonomous Region
Jinzhai Tianma national nature reserve	-0.05789	-0.109766	Anhui Province
Jiaozi Shan national nature reserve	0.068991	0.105013	Yunnan Province
Jiangle Longqishan national nature reserve	-0.040427	-0.064486	Fujian Province
Jiangxi Matoushan national nature reserve	-0.043457	-0.079968	Jiangxi Province
Jianfeng Ling national nature reserve	-0.028705	-0.060673	Hainan Province
Ji County Middle to Upper Proterozoic Strata Section national nature reserve	-0.008449	-0.016186	Tianjin Municipality
Jilin Jian national nature reserve	0.147513	0.235233	Jilin Province
Jilin Changbai Shan national nature reserve	0.076373	0.150117	Jilin Province

Hui River national nature reserve	-0.027737	0.157617	Inner Mongolia Autonomous Region
Hunchun Northeast Tiger national nature reserve	0.089082	0.149275	Jilin Province
Huangsang national nature reserve	-0.043336	-0.084555	Hunan Province
Huangnihe national nature reserve	0.146795	0.236464	Jilin Province
Huanglian Shan national nature reserve	0.004488	0.022462	Yunnan Province
Yellow River First Bend national nature reserve	0.148735	0.322938	Gansu Province
Yellow River Delta national nature reserve	-0.007883	-0.029222	Shandong Province
Huangbo Yuan national nature reserve	0.086239	0.106358	Shaanxi Province
Huagao Xi national nature reserve	-0.030832	-0.120943	Sichuan Province
Huashan national nature reserve	0.012258	-0.015993	Shaanxi Province
Huaping national nature reserve	-0.034039	-0.085084	Guangxi Zhuang Autonomous Region
Hua'e Shan national nature reserve	-0.019229	-0.063065	Sichuan Province
Huboliao national nature reserve	-0.000375	-0.028566	Fujian Province
Hunan Yangming Shan National Nature Reserve	-0.036369	-0.068396	Hunan Province
Hunan Shunhuang Shan national nature reserve	-0.068263	-0.118199	Hunan Province
Hubing Shan national nature reserve	-0.050983	-0.095707	Hunan Province
Huzhong national nature reserve	0.03025	0.251829	Heilongjiang Province
Honghu national nature reserve	-0.037338	-0.028312	Hubei Province
Honghe national nature reserve	0.193219	0.245645	Heilongjiang Province
Hongluo Shan national nature reserve	0.028409	0.005668	Liaoning Province
Hongxing Wetland national nature reserve	0.188514	0.335366	Heilongjiang Province
Hongjianapo national nature reserve	-0.046021	-0.095358	Shaanxi Province
Hengshui Lake national nature reserve	0.000412	-0.030179	Hebei Province
Heilongjiang Shuanghe national nature reserve	0.308361	0.435852	Heilongjiang Province
Heilongjiang Pingding Shan national nature reserve	0.063847	0.241339	Heilongjiang Province
Heilongjiang Fenghuang Shan national nature reserve	0.070613	0.07675	Heilongjiang Province

Heilongjiang Dongfanghong Wetland National Nature Reserve	0.078506	0.179645	Heilongjiang Province
Heilongjiang Daxiagu national nature reserve	0.124891	0.206947	Heilongjiang Province
Heicha Shan national nature reserve	0.066194	0.047119	Shaanxi Province
Henan Yellow River Wetland national nature reserve	-0.030805	-0.086982	Henan Province
Henan Dabie Shan national nature reserve	-0.048804	-0.084663	Henan Province
Henan Baotianman National Nature Reserve	-0.04068	-0.078113	Henan Province
Hebei Wuling Shan national nature reserve	0.067118	0.040704	Hebei Province
Hanzhong Red-crowned Crane national nature reserve	-0.019148	-0.064052	Shaanxi Province
Hanshan national nature reserve	0.216421	0.266688	Inner Mongolia Autonomous Region
Hancheng Huanglongshan Hemaji national nature reserve	-0.007571	-0.016723	Shaanxi Province
Hai Zi Shan national nature reserve	0.152451	0.304292	Sichuan Province
Haitang Shan national nature reserve	0.032855	-0.00805	Liaoning Province
Hatentsaohai national nature reserve	-0.08788	-0.220799	Inner Mongolia Autonomous Region
Hanasi national nature reserve	0.118461	0.239334	Xinjiang Uyghur Autonomous Region
Habahu national nature reserve	-0.064462	-0.142943	Ningxia Hui Autonomous Region
Guanshan national nature reserve	-0.053033	-0.080782	Jiangxi Province
Gutian Shan national nature reserve	-0.04429	-0.074196	Zhejiang Province
Gudigesitai national nature reserve	0.231853	0.285981	Inner Mongolia Autonomous Region
Ancient Coast and Wetland national nature reserve	-0.009025	-0.018765	Tianjin Municipality
Gongga Shan national nature reserve	0.124474	0.230462	Sichuan Province
Gongbierah River national nature reserve	0.294948	0.433141	Heilongjiang Province
Gexi Gou national nature reserve	0.129342	0.261146	Sichuan Province
Gaoligong Shan national nature reserve	0.055025	0.079127	Yunnan Province
Gaogesitai Hanwula national nature reserve	0.206927	0.24974	Inner Mongolia Autonomous Region
Ganjiang Source national nature reserve	-0.031291	-0.050959	Jiangxi Province
Gansu Qilian Shan national nature reserve	0.0261	0.119736	Gansu Province

Ganjiahu Saxaul Forest national nature reserve	-0.087549	-0.257953	Xinjiang Uyghur Autonomous Region
Gaha - Zecha national nature reserve	0.168809	0.329157	Gansu Province
Fujian Wuyi Shan national nature reserve	-0.040004	-0.083065	Fujian Province
Fujian Emei Peak national nature reserve	-0.051079	-0.097877	Fujian Province
Foping national nature reserve	0.036109	0.023697	Shaanxi Province
Funiu Shan national nature reserve	-0.011306	-0.04244	Henan Province
Fengtongzhai national nature reserve	0.044555	0.047898	Sichuan Province
Fenglin national nature reserve	0.099754	0.263645	Heilongjiang Province
Fangcheng Golden Flower Tea national nature reserve	-0.027999	-0.084759	Guangxi Zhuang Autonomous Region
Fanjing Shan national nature reserve	-0.029579	-0.07568	Guizhou Province
Encheng national nature reserve	-0.02217	-0.066341	Guangxi Zhuang Autonomous Region
Ertuoke Dinosaur Fossil Site national nature reserve	-0.071239	-0.188724	Inner Mongolia Autonomous Region
Ejina Populus euphratica Forest national nature reserve	-0.13675	-0.176803	Inner Mongolia Autonomous Region
Eerguna national nature reserve	0.033151	0.256039	Inner Mongolia Autonomous Region
Duoer national nature reserve	0.179574	0.285107	Gansu Province
Dobukuer national nature reserve	0.298149	0.375789	Heilongjiang Province
Dunhuang Yangguan national nature reserve	-0.203616	-0.293221	Gansu Province
Dunhuang Xihu national nature reserve	-0.174779	-0.227545	Gansu Province
Duhe Source national nature reserve	-0.015056	-0.061928	Hebei Province
Dongzhai national nature reserve	-0.068682	-0.112672	Henan Province
Dongfanghong Wetland national nature reserve	0.131037	0.184717	Heilongjiang Province
Dongting Lake national nature reserve	-0.034146	-0.027513	Hunan Province
Diaoluoshan national nature reserve	0.001459	-0.008549	Hainan Province
Danjiang Wetland national nature reserve	-0.087515	-0.137163	Henan Province
Dazhanhe Wetland national nature reserve	0.175547	0.328678	Heilongjiang Province
Dayao Shan national nature reserve	-0.024763	-0.03257	Guangxi Zhuang Autonomous Region

Da Xingan Ling Hanma national nature reserve	-0.088099	0.221067	Inner Mongolia Autonomous Region
Datong Beichuan River Source Area national nature reserve	0.065531	0.248729	Qinghai Province
Dashanbao Black-necked Crane national nature reserve	0.030043	0.039593	Yunnan Province
Dashah River national nature reserve	-0.031509	-0.080963	Guizhou Province
Dalao Ling national nature reserve	-0.032711	-0.0713	Hubei Province
Daheshan national nature reserve	0.016459	-0.012728	Inner Mongolia Autonomous Region
Dabie Shan national nature reserve	-0.076311	-0.141714	Hubei Province
Daba Shan national nature reserve	0.003985	-0.024809	Chongqing Municipality
Dalinor national nature reserve	0.196465	0.194449	Inner Mongolia Autonomous Region
Dalai Lake national nature reserve	-0.113272	0.132154	Inner Mongolia Autonomous Region
Cui Bei Wetland national nature reserve	0.198089	0.354476	Heilongjiang Province
Chonar River national nature reserve	0.315189	0.43611	Heilongjiang Province
Chongzuo White-headed Langur national nature reserve	-0.060813	-0.12463	Guangxi Zhuang Autonomous Region
Chishui Sago Palm national nature reserve	-0.035622	-0.1253	Guizhou Province
Qaidam Saxaul Forest national nature reserve	-0.069349	-0.150905	Qinghai Province
Chaoyu Zibagou national nature reserve	0.128777	0.249937	Tibet Autonomous Region
Chaqing Songduo White-lipped Deer national nature reserve	0.16483	0.312847	Sichuan Province
Chaigan Lake national nature reserve	0.013698	0.080735	Jilin Province
Cangshan Erhai national nature reserve	0.026682	0.038003	Yunnan Province
Boluo Lake national nature reserve	0.086657	0.098456	Jilin Province
Bila River national nature reserve	0.294398	0.362614	Inner Mongolia Autonomous Region
Beiji Village national nature reserve	0.08434	0.29869	Heilongjiang Province
Baoqing Qixinghe national nature reserve	0.083465	0.089093	Heilongjiang Province
Bangliang Gibbon national nature reserve	-0.062336	-0.126242	Guangxi Zhuang Autonomous Region
Baihua Shan national nature reserve	0.032259	-0.004858	Beijing Municipality
Baiyun Shan national nature reserve	-0.065639	-0.113295	Hunan Province

Baiyin Aobao national nature reserve	0.242385	0.289223	Inner Mongolia Autonomous Region
Baishui River national nature reserve	0.065208	0.072452	Gansu Province
Baishui River national nature reserve	0.022971	0.029753	Sichuan Province
Baishi Jiasi national nature reserve	0.094973	0.131349	Liaoning Province
Baishan Père Davids Deer national nature reserve	0.103314	0.161698	Jilin Province
Baimaxueshan national nature reserve	0.099507	0.162374	Yunnan Province
Baihe River national nature reserve	0.191421	0.275386	Sichuan Province
Bayin Brug national nature reserve	-0.149506	-0.196178	Xinjiang Uyghur Autonomous Region
Balikun Shan national nature reserve	0.10343	0.162654	Xinjiang Uyghur Autonomous Region
Badong Golden Snub-nosed Monkey national nature reserve	-0.031404	-0.066595	Hubei Province
Badagong Shan national nature reserve	-0.045978	-0.090345	Hunan Province
Bacha Island national nature reserve	0.186786	0.253884	Heilongjiang Province
Anxi Extreme Arid Desert national nature reserve	-0.118514	-0.245838	Gansu Province
Annanba Wild Camel national nature reserve	-0.123177	-0.202319	Gansu Province
Aibi Lake Wetland national nature reserve	-0.085714	-0.255294	Xinjiang Uyghur Autonomous Region
Alukorqin national nature reserve	-0.021156	-0.003946	Inner Mongolia Autonomous Region
Altay Kekesu Wetland national nature reserve	-0.08339	-0.226561	Xinjiang Uyghur Autonomous Region
Altun Shan national nature reserve	-0.119756	-0.160836	Xinjiang Uyghur Autonomous Region

Table S3 Alien woody plant species identified as high-risk (⩾10 % above the mean invasion probability) in China's national nature reserves under current and future climate scenarios (Current, SSP245, SSP585).

Species	current	ssp245	ssp585	Species	current	ssp245	ssp585
Pinus thunbergii	+	+		*Juglans cinerea*			
Quercus robur	+	+	+	*Casuarina cunninghamiana*			
Sorbaria tomentosa	+	+	+	*Pimenta dioica*			
Salix alba	+	+	+	*Callistemon viminalis*			
Populus tremuloides	+	+	+	*Hevea brasiliensis*			
Acer negundo	+	+	+	*Pinus elliottii*			
Ribes aureum	+	+	+	*Cedrela odorata*			
Robinia pseudoacacia	+	+	+	*Rosa pendulina*			
Pseudotsuga menziesii	+	+	+	*Picea sitchensis*			
Populus nigra	+	+	+	*Helianthemum ledifolium*			
Ribes nigrum	+	+	+	*Abies nordmanniana*			
Amorpha fruticosa	+	+	+	*Acacia melanoxylon*			
Populus deltoides	+	+	+	*Pinus parviflora*			
Pinus ponderosa	+	+	+	*Genista pilosa*			
Betula pubescens	+	+	+	*Gliricidia sepium*			
Populus balsamifera	+	+	+	*Rhododendron ponticum*			
Pinus contorta	+	+	+	*Tabebuia rosea*			
Calluna vulgaris	+	+	+	*Eucalyptus grandis*			
Dalbergia sissoo	+	+	+	*Pinus canariensis*			
Alnus glutinosa	+	+	+	*Pinus taeda*			
Fraxinus pennsylvanica	+	+	+	*Mimosa pigra*			
Ribes multiflorum	+	+	+	*Tipuana tipu*			

Berberis vulgaris	+	+	+	*Genista germanica*
Styphnolobium japonicum	+	+	+	*Phytolacca dioica*
Cedrus deodara	+	+	+	*Pinus luchuensis*
Acer platanoides	+	+	+	*Rhododendron ferrugineum*
Prunus domestica	+	+	+	*Fraxinus uhdei*
Cytisus scoparius	+	+	+	*Borassus flabellifer*
Lantana camara	+	+	+	*Acacia mangium*
Pinus strobus	+	+	+	*Pinus patula*
Picea abies	+	+	+	*Eucalyptus urophylla*
Acer pseudoplatanus	+	+	+	*Eucalyptus tereticornis*
Aesculus hippocastanum	+	+	+	*Pinus oocarpa*
Ulmus minor	+	+	+	*Pinus pinea*
Ligustrum vulgare	+	+	+	*Pachira aquatica*
Ficus religiosa	+	+	+	*Corymbia citriodora*
Fagus sylvatica	+	+	+	*Ptychosperma macarthurii*
Ribes uva-crispa	+	+	+	*Pinus radiata*
Ulmus americana	+	+	+	*Reutealis trisperma*
Quercus rubra	+	+	+	*Pinus caribaea*
Cryptomeria japonica	+	+	+	*Psidium guineense*
Arenga pinnata	+	+	+	*Rubus moluccanus*
Fraxinus excelsior	+	+	+	*Triumfetta bogotensis*
Lonicera xylosteum	+	+	+	*Abies procera*
Philadelphus coronarius	+	+	+	*Corymbia torelliana*
Salix nigra	+	+	+	*Staphylea pinnata*
Tilia cordata	+	+	+	*Juglans ailanthifolia*
Spartium junceum	+	+	+	*Cecropia peltata*

Cornus sanguinea	+	+	+	*Cytisus nigricans*
Berberis thunbergii	+	+	+	*Alnus rubra*
Fraxinus americana	+	+	+	*Abies amabilis*
Ficus benghalensis		+	+	*Senna spectabilis*
Castanea sativa	+	+	+	*Mammea americana*
Catalpa speciosa	+	+	+	*Eucalyptus saligna*
Callistemon rigidus	+	+	+	*Rhododendron hirsutum*
Juglans nigra	+	+	+	*Eucalyptus cinerea*
Eucalyptus robusta	+	+	+	*Myroxylon balsamum*
Persea americana	+	+	+	*Cotoneaster simonsii*
Pinus nigra	+	+	+	*Heterotis rotundifolia*
Juniperus virginiana	+	+	+	*Polygala myrtifolia*
Ribes alpinum	+	+	+	*Cordia alliodora*
Juniperus occidentalis	+	+	+	*Triplaris americana*
Azadirachta indica		+	+	*Sonneratia apetala*
Pyracantha coccinea	+	+	+	*Acacia decurrens*
Schinus molle		+	+	*Berberis darwinii*
Cassia fistula		+	+	*Eucalyptus pellita*
Physocarpus opulifolius	+	+	+	*Eucalyptus viminalis*
Genista tinctoria		+	+	*Pinus glabra*
Spiraea alba	+	+	+	*Daboecia cantabrica*
Carpinus betulus	+	+	+	*Acacia implexa*
Casuarina equisetifolia		+	+	*Grevillea banksii*
Larix decidua	+	+	+	*Hura crepitans*
Albizia lebbeck			+	*Cinchona pubescens*
Fraxinus angustifolia	+	+	+	*Eucalyptus benthamii*

Quercus petraea		+	+	*Ptychosperma elegans*	+
Pinus banksiana	+	+	+	*Artocarpus altilis*	
Myrica gale	+	+	+	*Abies sachalinensis*	
Acaciella glauca		+	+	*Ficus rubiginosa*	
Tilia platyphyllos		+	+	*Melicoccus bijugatus*	
Delonix regia			+	*Sterculia apetala*	
Pinus monticola	+	+	+	*Medicago arborea*	
Artocarpus heterophyllus		+	+	*Schizolobium parahyba*	
Abies alba		+	+	*Acacia fimbriata*	
Ulex europaeus			+	*Syzygium paniculatum*	
Amelanchier ovalis	+	+	+	*Senna multijuga*	
Eucalyptus globulus			+	*Acacia holosericea*	
Annona squamosa			+	*Syzygium malaccense*	
Spiraea douglasii	+	+	+	*Acacia adunca*	
Aleurites moluccanus			+	*Berberis microphylla*	
Chamaecyparis lawsoniana			+	*Erythrina poeppigiana*	
Larix kaempferi			+	*Atriplex nummularia*	
Cupressus sempervirens			+	*Allocasuarina littoralis*	
Washingtonia robusta			+	*Acacia podalyriifolia*	
Tectona grandis			+	*Hakea salicifolia*	
Averrhoa carambola				*Roystonea oleracea*	
Helianthemum apenninum				*Corymbia maculata*	
Galphimia glauca				*Elaeis guineensis*	
Gomphocarpus fruticosus				*Amelanchier lamarckii*	
Abies grandis				*Dovyalis caffra*	
Salix repens				*Acacia paradoxa*	

Tephrosia candida	+	+	*Acacia elata*
Quercus coccifera			*Pentadesma butyracea*
Garcia nutans			*Archontophoenix cunninghamiana*
Grevillea robusta			*Eucalyptus sideroxylon*
Pinus rigida			*Chamaecytisus ratisbonensis*
Quercus cerris			*Callistemon linearis*
Cinnamomum verum			*Melianthus major*
Jacaranda mimosifolia			*Eucalyptus megacornuta*
Eucalyptus camaldulensis			*Voacanga africana*
Hippocrepis emerus			*Triplaris cumingiana*
Tamarindus indica			*Eucalyptus gunnii*
Solanum anguivi			*Eucalyptus nitens*
Pinus pinaster			*Eucalyptus cloeziana*
Spathodea campanulata			*Copaifera officinalis*
Manilkara zapota			*Casuarina glauca*
Pinus halepensis			*Phytelephas macrocarpa*
Ulmus laevis			*Eucalyptus botryoides*
Genista sagittalis			*Dillenia suffruticosa*
Castanea crenata			*Eucalyptus gomphocephala*
Washingtonia filifera			*Metrosideros excelsa*
Cochlospermum religiosum			*Eucalyptus brassiana*
Senna siamea			*Eucalyptus cladocalyx*
Rhamnus alaternus			*Billardiera heterophylla*
Symphoricarpos orbiculatus			*Melaleuca hypericifolia*
Eucalyptus diversicolor			*Acacia iteaphylla*
Eucalyptus lehmannii			

“+” represents species with a probability of invasive trees in potential national parks being 10% higher than the probability of invasive trees in national nature reserves.

Supplementary Material S1 GBIF Data Citations

GBIF.org (30 June 2023) GBIF Occurrence Download https://doi.org/10.15468/dl.fyy9xv

GBIF.org (30 June 2023) GBIF Occurrence Download https://doi.org/10.15468/dl.ebfwve

GBIF.org (30 June 2023) GBIF Occurrence Download https://doi.org/10.15468/dl.sayetr

GBIF.org (30 June 2023) GBIF Occurrence Download https://doi.org/10.15468/dl.qg4czc

GBIF.org (01 July 2023) GBIF Occurrence Download https://doi.org/10.15468/dl.tbmybx

GBIF.org (01 July 2023) GBIF Occurrence Download https://doi.org/10.15468/dl.taeesw

GBIF.org (01 July 2023) GBIF Occurrence Download https://doi.org/10.15468/dl.tad38m

GBIF.org (01 July 2023) GBIF Occurrence Download https://doi.org/10.15468/dl.x7jxc9

GBIF.org (01 July 2023) GBIF Occurrence Download https://doi.org/10.15468/dl.x7jxc9

GBIF.org (01 July 2023) GBIF Occurrence Download https://doi.org/10.15468/dl.ckrchy

GBIF.org (01 July 2023) GBIF Occurrence Download https://doi.org/10.15468/dl.ckrchy

GBIF.org (01 July 2023) GBIF Occurrence Download https://doi.org/10.15468/dl.8mv24q

GBIF.org (01 July 2023) GBIF Occurrence Download https://doi.org/10.15468/dl.3de5sa

GBIF.org (01 July 2023) GBIF Occurrence Download https://doi.org/10.15468/dl.sez87y

GBIF.org (01 July 2023) GBIF Occurrence Download https://doi.org/10.15468/dl.uubprd

GBIF.org (01 July 2023) GBIF Occurrence Download https://doi.org/10.15468/dl.jghj6s

GBIF.org (02 July 2023) GBIF Occurrence Download https://doi.org/10.15468/dl.kd3uxh

GBIF.org (02 July 2023) GBIF Occurrence Download https://doi.org/10.15468/dl.chykvr

GBIF.org (02 July 2023) GBIF Occurrence Download https://doi.org/10.15468/dl.qxqb87

GBIF.org (02 July 2023) GBIF Occurrence Download https://doi.org/10.15468/dl.nrytp2

GBIF.org (02 July 2023) GBIF Occurrence Download https://doi.org/10.15468/dl.fj7afq

GBIF.org (02 July 2023) GBIF Occurrence Download https://doi.org/10.15468/dl.rzhmsy

GBIF.org (02 July 2023) GBIF Occurrence Download https://doi.org/10.15468/dl.rzhmsy

GBIF.org (02 July 2023) GBIF Occurrence Download https://doi.org/10.15468/dl.kttkmu

GBIF.org (02 July 2023) GBIF Occurrence Download https://doi.org/10.15468/dl.25csz7

GBIF.org (02 July 2023) GBIF Occurrence Download https://doi.org/10.15468/dl.bpd822

GBIF.org (02 July 2023) GBIF Occurrence Download https://doi.org/10.15468/dl.98ndzw

GBIF.org (02 July 2023) GBIF Occurrence Download https://doi.org/10.15468/dl.8dnaug
